# The association between dietary vitamins and the risk of sarcopenia in adults aged 20–59: a study based on the NHANES database

**DOI:** 10.3389/fnut.2025.1535190

**Published:** 2025-03-26

**Authors:** Yu Qiu, Fengyi Zhao, Xiyin Zheng, Xiaohua Wang

**Affiliations:** ^1^The Department of Clinical Nutrition, Nantong First People's Hospital, Nantong, Jiangsu, China; ^2^The Department of Clinical Nutrition, Affiliated Hospital 2 of Nantong University, Nantong, Jiangsu, China; ^3^The Department of Clinical Nutrition, Nantong Hospital of Renji Hospital Affiliated to Shanghai Jiao Tong University School of Medicine, Nantong, Jiangsu, China; ^4^The Department of Endocrinology and Metabolism, Nantong First People's Hospital, Nantong, Jiangsu, China; ^5^The Department of Endocrinology and Metabolism, Affiliated Hospital 2 of Nantong University, Nantong, Jiangsu, China; ^6^The Department of Endocrinology and Metabolism, Nantong Hospital of Renji Hospital Affiliated to Shanghai Jiao Tong University School of Medicine, Nantong, Jiangsu, China

**Keywords:** diet, nutrition, vitamin, skeletal muscle mass, sarcopenia

## Abstract

**Background:**

Sarcopenia has emerged as a global health concern, but the association between dietary vitamin levels and sarcopenia is not elucidated. This study aims to shed light on the link of 11 dietary vitamins to the risk of sarcopenia in adults at the age of 20–59.

**Methods:**

This cross-sectional study encompassed 2011–2018 data from the National Health and Nutrition Examination Survey (NHANES) for adults aged 20–59. Sarcopenia was defined through the appendicular lean mass to body mass index ratio calculated via Dual-Energy X-ray Absorptiometry (DXA), with sarcopenia determined as a ratio of <0.789 for the male and <0.512 for the female. Multivariate weighted logistic regression assisted in assessing the connection of dietary vitamins with sarcopenia, with results presented as odds ratios (ORs) and 95% confidence intervals (CIs). The dose-response association of various vitamins with sarcopenia was visualized through restricted cubic splines (RCS). Subgroup analyses were carried out to examine the consistency of the aforementioned associations. Sensitivity analysis was performed utilizing propensity score matching (PSM) to adjust for confounding factors and enhance the robustness of the results.

**Results:**

Among the 7,864 participants, 677 (8.6%) had sarcopenia, and 7,187 (91.4%) did not. Multivariate weighted logistic regression and RCS analyses indicated that higher intakes of VA, VB1, VB2, VB3, VB6, VB9, VB12, VC, VE, and VK were notably linked to a lowered risk of sarcopenia (*P* < 0.05). Among these, VA, VC, and VE exhibited a non-linear negative association with sarcopenia risk (*P* for non-linear < 0.05), while VB1, VB2, VB3, VB6, VB9, VB12, and VK exhibited a linear negative association (*P* for non-linear > 0.05). Subgroup analysis yielded largely consistent results. After confounding factors were adjusted through PSM, the results suggest that the intake of VA, VB2, VB6, and VC remains significantly associated with a lowered risk of sarcopenia (*P* < 0.05).

**Conclusion:**

Higher dietary levels of VA, VB2, VB6, and VC are significantly related to a lower livelihood of sarcopenia. These findings provide new evidence and insights for early dietary interventions aimed at preventing sarcopenia.

## 1 Introduction

Sarcopenia refers to the progressive loss of skeletal muscle mass and physical function (muscle strength or performance) as a person ages and it becomes more prevalent (1). It can lead to plenty of adverse outcomes in the old, such as impaired physical function, diminished quality of life, and elevated risk of mortality, severely impacting their health and life, thereby imposing a substantial strain on families and society. An article from Europe predicts that, over the next 30 years, the number of patients with sarcopenia will increase sharply, with the prevalence of sarcopenia among the old rising from 20.2% in 2016 to 22.3% by 2045 (2). Therefore, sarcopenia poses a major challenge to public health (3–5), with a growing trend of increased healthcare resource utilization and higher medical costs (6). Although sarcopenia has gained considerable attention from most clinicians, a universally accepted and clinically applicable definition remains lacking on an international scale (7). The European Working Group on Sarcopenia in Older People (EWGSOP) defines sarcopenia as a syndrome characterized by the progressive loss of skeletal muscle mass and strength. It is categorized into three stages based on severity: “pre-sarcopenia,” “sarcopenia,” and “severe sarcopenia.” The pre-sarcopenia stage is characterized by low muscle mass but without any impact on muscle strength or physical performance. The sarcopenia stage involves low muscle mass along with either low muscle strength or poor physical performance. Severe sarcopenia is defined as the stage in which all three criteria are met: low muscle mass, low muscle strength, and poor physical performance (8). In addition, the Foundation for the National Institutes of Health (FNIH) defines sarcopenia based on declines in muscle mass, muscle strength, and muscle function, providing corresponding cut-off values based on data from U.S. citizens (9). Its etiology and pathogenesis are multifactorial, including factors such as malnutrition, reduced physical activity, chronic inflammation, and oxidative stress (10). The decline in appetite and digestive function in older adults often leads to insufficient intake or absorption of protein, energy, and trace elements, resulting in malnutrition, which is closely associated with sarcopenia (11). Therefore, nutritional intervention serves as a cornerstone in sarcopenia prevention and treatment. The focus of current research is primarily on the role of macronutrients, such as protein energy metabolism and essential amino acids and their metabolites, in the prevention of sarcopenia. However, large-scale studies exploring the association between vitamins, as micronutrients, and sarcopenia are relatively scarce.

Vitamins are a class of trace organic substances that must be obtained from food and play critical roles in growth, metabolism, and development. They are classified into fat-soluble vitamins, like VA, VD, VE, and VK, and water-soluble vitamins such as VB and VC. It has been demonstrated that multiple vitamins are closely related to sarcopenia (12–14), suggesting that vitamins could serve as potential targets for sarcopenia intervention. Sarcopenia begins to develop gradually in midlife and is accompanied by the deterioration and imbalance of various physiological systems (15). Proper vitamin intake from early stages may lower the risk of muscle loss (16). However, there has yet to be a comprehensive, systematic investigation into the influence of different dietary vitamin intakes on sarcopenia, and existing studies on specific individual vitamins have yielded varying, sometimes contradictory conclusions (17–20).

Therefore, this study delves into the connection of dietary vitamin intake with sarcopenia in Americans between 20 and 59 years old with 2011–2018 data from the National Health and Nutrition Examination Survey (NHANES) and seeks to provide effective nutritional strategies for preventing sarcopenia in adulthood.

## 2 Methods

### 2.1 Study population

NHANES is a large-scale cross-sectional survey carried out by the National Center for Health Statistics (NCHS) and the American Centers for Disease Control and Prevention (CDC). It adopts a complicated stratified multistage probability sampling approach to gather health and nutrition information from a nationally representative sample of people who are not institutionalized. It seeks to investigate risk factors for diseases and assess the prevalence of major health conditions. Since 1999, it has been released biennially with continuously collected data. The study protocol was approved by the NCHS Institutional Review Board, and informed consent was gained from the participants. The utilized data were from four cycles of NHANES (2011–2018), comprising 7,864 participants. The dataset is publicly available at https://wwwn.cdc.gov/nchs/nhanes/. This database adheres strictly to ethical standards to safeguard participants' rights and privacy. Additional ethical information is detailed at https://www.cdc.gov/nchs/nhanes/irba98.htm.

**Figure 1:**
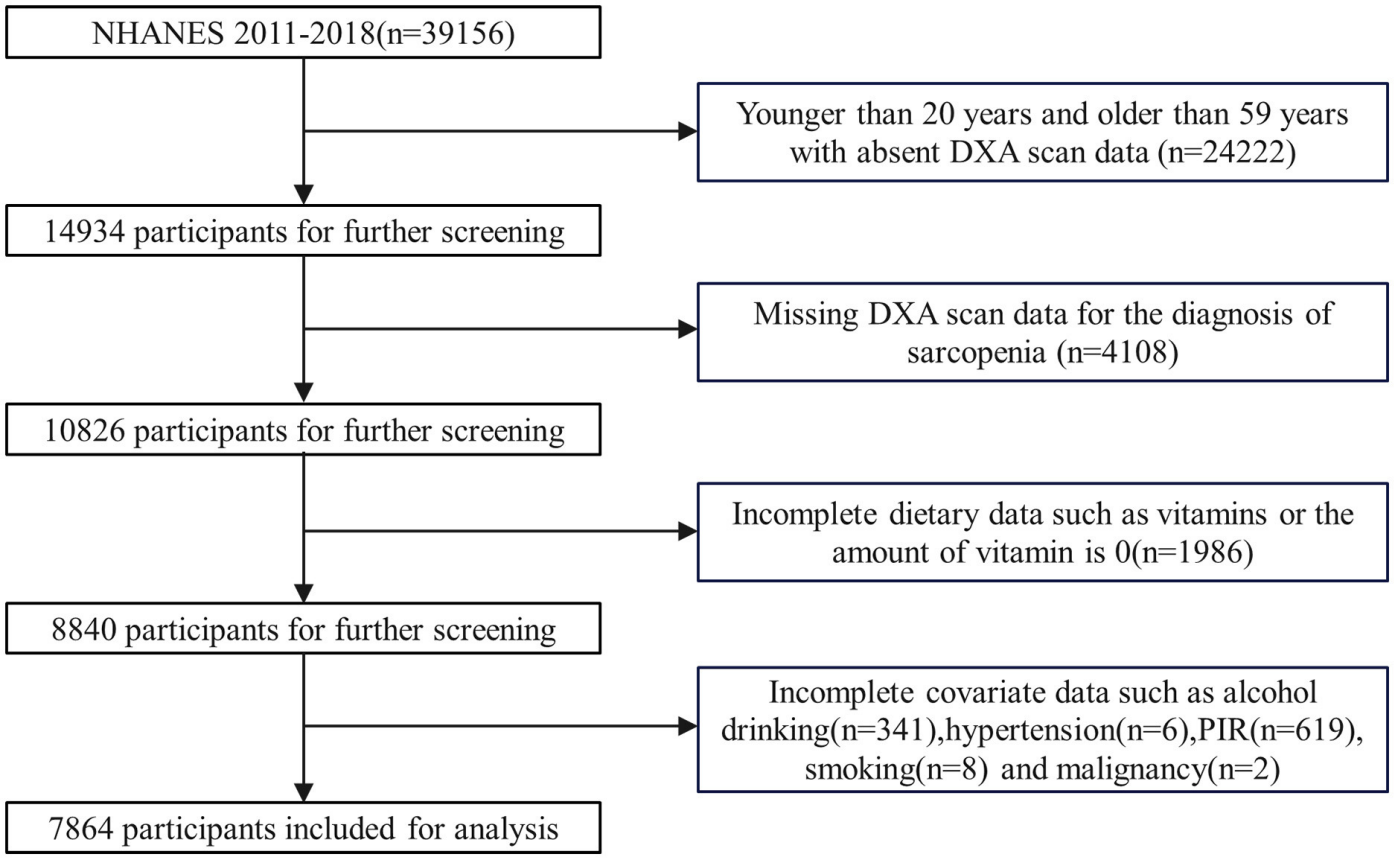
Flow chart for the selection of participants in the cohort study. DXA, dual-energy x-ray absorptiometry; PIR, poverty income ratio.

The inclusion criteria for this study were: participants with recorded Dual-energy X-ray Absorptiometry (DXA) scan results, key outcome variables for the diagnosis of sarcopenia, and complete dietary data, including vitamin intake, as well as complete covariate data. The exclusion criteria were: participants who did not undergo a DXA scan and did not have key outcome variables for the diagnosis of sarcopenia, and had incomplete or no dietary data (including vitamins), and incomplete covariate data (Figure 1).

### 2.2 Dietary vitamins

Nutritional data were gathered after two 24-h dietary recall interviews. Participants were asked about the type and amount of foods, beverages, and dietary supplements had in the past 24 h. The first dietary recall was performed in person at the Mobile Examination Center (MEC), and the second was carried out by phone 3 to 10 days later. To obtain more accurate estimates of the nutrients in the foods, beverages, and supplements consumed, trained interviewers used the Automated Multiple-Pass Method (AMPM) system (http://www.ars.usda.gov/nea/bhnrc/fsrg) for diet data collection. This interview system assisted by computer employs a standardized five-step method to assess current dietary intake and minimize underreporting. The dietary vitamins extracted for this study encompass VA, VB1, VB2, VB3, VB6, VB9, VB12, VC, VD, VE, and VK.

### 2.3 Definition of sarcopenia

In adherence to the recommended sarcopenia diagnosis criteria of the Foundation for the National Institutes of Health (FNIH) (9), as well as previous studies (21, 22), sarcopenia is defined by the ratio of appendicular lean mass (ALM) to body mass index (BMI). The thresholds for sarcopenia are set at ALM/BMI values of < 0.789 for males and < 0.512 for females. ALM measurements are performed via Dual-Energy X-ray Absorptiometry (DXA) by trained and certified radiologic technicians. These measurements are obtained through a Hologic Discovery Model A densitometer (Hologic, Inc., Bedford, Massachusetts) with Apex 3.2. The DXA protocol is in the Body Composition Procedures Manual and available at https://www.cdc.gov/nchs/nhanes/index.htm.

### 2.4 Covariates

In our study, a standard interview questionnaire was utilized for gathering information on gender, age, ethnicity, education, marital status, poverty-to-income ratio (PIR), hypertension and malignancy history, alcohol consumption, smoking, and physical activity. BMI (kg/m^2^) was obtained through anthropometric measurements, while diabetes-related information was gathered through questionnaires and laboratory tests. Ethnicity was classified as Mexican American, non-Hispanic White, non-Hispanic Black, or Other. Education was divided into less than high school, high school or equivalent, and college or higher. Marital status was grouped as married or cohabitating, widowed, divorced or separated, and never married. Smoking involved non-smokers, former smokers (individuals who had smoked 100 or more cigarettes and quit), and current smokers (those who had smoked over 100 cigarettes and are smoking). Alcohol consumption was divided into drinkers and non-drinkers based on whether the individual consumed 12 or more alcoholic beverages in the past year. Physical activity was measured in metabolic equivalent (MET)-minutes every week. The diabetes diagnosis standards include: (1) being informed by a doctor or healthcare professional of having diabetes, (2) a hemoglobin A1c (HbA1c) level ≥6.5 mmol/L, (3) a fasting blood glucose level (FGLU) ≥7.0 mmol/L, (4) current use of anti-diabetic medication, or (5) current use of insulin.

### 2.5 Statistical analysis

Our statistical analyses accounted for the intricate, multi-stage, tiered, and clustered sampling of NHANES. Sample weights were appropriately allocated based on NHANES guidelines, with the WTDR2D weighting applied to combine samples from four distinct survey cycles. Participants were categorized into the sarcopenia group and the non-sarcopenia group. Categorical variables are presented as unweighted n (weighted %), and statistical comparisons were performed using the Rao-Scott chi-square test. Continuous variables, after the Anderson-Darling test, were found to follow non-normal distributions and are presented as medians (interquartile range), with comparisons made via the Mann-Whitney U test. Weighted logistic regression helped us examine the link of dietary vitamin intake to sarcopenia risk, with results displayed as ORs and 95% CIs. Three logistic regression models were constructed: Model 1 (unadjusted), Model 2 (adjusted for gender, age, ethnicity, education, marital status, PIR, and BMI), and Model 3 (adjusted for the variables in Model 2, history of diabetes, hypertension and malignancy, smoking, alcohol consumption, as well as physical activity. An RCS model assisted in visualizing the dose-response association between vitamin levels and sarcopenia. Subgroup analyses were conducted in terms of gender, age, ethnicity, educational level, marital status, diabetes, hypertension, malignancy, drinking, and smoking to assess the stability of the relationship between vitamins and sarcopenia. PSM was employed in the sensitivity analysis to assess the robustness of the outcomes. All analyses were conducted with the help of R. A two-tailed *p*-value < 0.05 signified statistical significance.

## 3 Results

### 3.1 Baseline characteristics of the study population

Seven thousand eight hundred and sixty four participants aged 20–59 were encompassed, with the majority being non-Hispanic white. The male-to-female ratio was approximately equal. They were classified as the sarcopenia group (*n* = 677) and the non-sarcopenia group (*n* = 7,187). The former group was older and had lower household income, higher BMI, and prevalence of diabetes and hypertension, as well as less alcohol consumption and physical activity (*P* < 0.05). Statistically significant differences also existed between them in ethnicity and educational level distribution (*P* < 0.05). Other baseline characteristics are presented in Table 1. The vitamin intake levels between the two groups showed statistically significant differences (*P* < 0.05), except for VD (*P* = 0.08).

**Table 1:** Basic characteristics of participants by sarcopenia in NHANES 2011–2018.

**Variables**	**Overall *N* = 7,864**	**Non-sarcopenia participants *N* = 7,187**	**Sarcopenia participants *N* = 677**	***P*-value**
**Gender**				0.2
Male	3,822 (50%)	3,489 (50%)	333 (54%)	
Female	4,042 (50%)	3,698 (50%)	344 (46%)	
Age	39 (29,50)	39 (29,50)	46 (34,55)	< 0.001
**Ethnicity**				< 0.001
Mexican American	1,083 (10%)	861 (9.1%)	222 (25%)	
Non-Hispanic White	2,947 (62%)	2,744 (63%)	203 (51%)	
Non-Hispanic Black	1,680 (11%)	1,632 (12%)	48 (3.4%)	
Other Ethnicity	2,154 (17%)	1,950 (16%)	204 (21%)	
**Education**				< 0.001
Less than high school diploma	1,218 (11%)	1,038 (10%)	180 (20%)	
High school diploma/equivalent	1,701 (22%)	1,516 (21%)	185 (29%)	
College or above	4,945 (67%)	4,633 (69%)	312 (51%)	
**Marriage**				0.072
Married/cohabitation	4,717 (61%)	4,291 (61%)	426 (63%)	
Widow/divorce/separation	1,090 (13%)	975 (13%)	115 (17%)	
Unmarried	2,057 (25%)	1,921 (26%)	136 (21%)	
PIR	2.97 (1.40,5.00)	3.05 (1.45,5.00)	1.97 (1.06,3.75)	< 0.001
BMI (kg/m^2^)	28 (24,32)	27 (24,32)	34 (30,40)	< 0.001
Log (VA) (mcg)	6.27 (5.81,6.70)	6.29 (5.83,6.72)	6.03 (5.52,6.50)	< 0.001
Log (VB1) (mg)	0.54 (0.19,0.93)	0.55 (0.20,0.94)	0.43 (0.12,0.77)	0.005
Log (VB2) (mg)	0.79 (0.44,1.20)	0.79 (0.45,1.22)	0.63 (0.26,1.01)	< 0.001
Log (VB3) (mg)	3.34 (3.00,3.67)	3.34 (3.00,3.69)	3.21 (2.86,3.54)	< 0.001
Log (VB6) (mg)	0.83 (0.44,1.34)	0.85 (0.45,1.36)	0.67 (0.30,1.02)	< 0.001
Log (VB9) (mcg)	6.34 (5.92,6.82)	6.36 (5.93,6.84)	6.25 (5.80,6.65)	< 0.001
Log (VB12) (mcg)	1.80 (1.17,2.53)	1.81 (1.18,2.55)	1.61 (1.06,2.22)	0.002
Log (VC) (mg)	4.38 (3.56,5.02)	4.40 (3.57,5.03)	4.12 (3.42,4.81)	< 0.001
Log (VD) (mcg)	1.65 (0.81,2.65)	1.66 (0.83,2.66)	1.49 (0.62,2.48)	0.080
Log (VE) (mg)	2.09 (1.72,2.47)	2.11 (1.73,2.48)	1.93 (1.56,2.25)	< 0.001
Log (VK) (mcg)	4.49 (3.97,5.03)	4.52 (3.99,5.05)	4.25 (3.74,4.76)	< 0.001
Ratio of Diabetes	847 (8.1%)	693 (7.0%)	154 (22%)	< 0.001
Ratio of Hypertension	1,860 (22%)	1,636 (21%)	224 (38%)	< 0.001
Ratio of Malignancy	333 (5.3%)	289 (5.1%)	44 (8.1%)	0.088
Ratio of Alcohol drinking	6,193 (83%)	5,720 (84%)	473 (75%)	< 0.001
**Smoking status**				0.6
Never	4,799 (60%)	4,373 (60%)	426 (61%)	
Former	1,344 (19%)	1,214 (19%)	130 (20%)	
Current	1,721 (21%)	1,600 (21%)	121 (19%)	
PAM (MET-min/week)	1,920 (480,5,720)	1,920 (480,5,760)	1,200 (0,3,960)	< 0.001

PIR, poverty income ratio; BMI, body mass index; VA, vitamin A; VB1, vitamin B1;VB2, vitamin B2;VB3, vitamin B3;VB6, vitamin B6; VB9, vitamin B9; VB12, vitamin B12; VC, vitamin C; VD, vitamin D; VE, vitamin E; VK, vitamin K; MET, metabolic equivalent of task; PAM, physical activity MET. All continuous variables deviated [IQR: P50 (P25, P75)]; categorical variables were denoted as unweighted n (weighted%). Categorical and continuous variables were compared via chi-squared and Wilcoxon rank-sum tests with Rao & Scott's correction for complex surveys.

### 3.2 Association between vitamin intake and sarcopenia risk

The association between vitamin intake levels and sarcopenia risk is shown in Table 2. Each vitamin was treated as a categorical variable, and the associations between 11 vitamins and the risk of sarcopenia were examined through three weighted logistic regression models. After adjusting for covariates, vitamins A, B1, B2, B3, B6, B9, B12, C, E, and K showed negative associations with sarcopenia (OR < 1, *P* < 0.05), while VD did not show a significant relation to sarcopenia risk (*P* > 0.05). Specifically, in contrast to the lowest quartile (Q1), the third quartile (Q3) levels of vitamins A, B1, B2, B3, B6, B9, B12, C, E, and K were all negatively correlated with the risk of sarcopenia [(VA, OR = 0.486, 95%CI:0.334–0.708, *P* < 0.001), (VB1, *OR* = 0.582, 95%CI:0.372–0.911, *P* = 0.019), (VB2, *OR* = 0.442, 95%CI:0.294–0.663, *P* < 0.001), (VB3, *OR* = 0.569, 95%CI:0.377–0.858, *P* = 0.008), (VB6, *OR* = 0.529, 95%CI:0.339–0.824, *P* = 0.006), (VB9, *OR* = 0.620, 95%CI:0.407–0.942, *P* = 0.026), (VB12, *OR* = 0.635, 95%CI:0.449–0.898, *P* = 0.011), (VC, *OR* = 0.664, 95%CI:0.450–0.980, *P* = 0.04), (VE, *OR* = 0.519, 95%CI:0.345–0.780, *P* = 0.002), and (VK, *OR* = 0.564, 95%CI:0.359–0.887, *P* = 0.014)].

**Table 2:** Multi-variate adjusted odds ratios (95% CIs) of risks of Sarcopenia in relation to different dietary vitamins levels among participants in NHANES 2011–2018.

**Variables**	**The range of dietary vitamin levels**	**Model 1**	***P*-value**	**Model 2**	***P*-value**	**Model 3**	***P*-value**
**Log (VA) (mcg)**							
Q1	−0.69–5.72	1		1		1	
Q2	5.72–6.64	0.541 (0.416,0.702)	< 0.001	0.505 (0.375,0.680)	< 0.001	0.501 (0.375,0.671)	< 0.001
Q3	6.64–8.90	0.454 (0.317,0.652)	< 0.001	0.489 (0.336,0.712)	< 0.001	0.486 (0.334,0.708)	< 0.001
p for trend			< 0.001		< 0.001		< 0.001
**Log (VB1) (mg)**							
Q1	−3.05–0.16	1		1		1	
Q2	0.16–0.90	0.913 (0.689,1.209)	0.519	0.858 (0.629,1.171)	0.328	0.848 (0.616,1.169)	0.306
Q3	0.90–6.46	0.592 (0.406,0.861)	0.007	0.590 (0.378,0.919)	0.021	0.582 (0.372,0.911)	0.019
p for trend			0.006		0.019		0.017
**Log (VB2) (mg)**							
Q1	−2.78–0.36	1		1		1	
Q2	0.36–1.12	0.636 (0.477,0.848)	0.003	0.607 (0.428,0.860)	0.006	0.624 (0.441,0.884)	0.009
Q3	1.12–5.36	0.459 (0.315,0.670)	< 0.001	0.425 (0.284,0.637)	< 0.001	0.442 (0.294,0.663)	< 0.001
p for trend			< 0.001		< 0.001		< 0.001
**Log (VB3) (mg)**							
Q1	0.32–2.96	1		1		1	
Q2	2.96–3.64	0.826 (0.622,1.097)	0.183	0.737 (0.536,1.012)	0.059	0.736 (0.533,1.017)	0.063
Q3	3.64–7.83	0.561 (0.386,0.817)	0.003	0.547 (0.363,0.823)	0.005	0.569 (0.377,0.858)	0.008
p for trend			0.002		0.004		0.007
**Log (VB6) (mg)**							
Q1	−2.59–0.39	1		1		1	
Q2	0.39–1.26	0.788 (0.590,1.052)	0.105	0.807 (0.567,1.149)	0.228	0.812 (0.568,1.160)	0.245
Q3	1.26–9.21	0.469 (0.322,0.683)	< 0.001	0.527 (0.339,0.820)	0.005	0.529 (0.339,0.824)	0.006
p for trend			< 0.001		0.005		0.005
**Log (VB9) (mcg)**							
Q1	2.60–5.89	1		1		1	
Q2	5.89–6.78	0.799 (0.592,1.080)	0.142	0.749 (0.536,1.048)	0.09	0.738 (0.526,1.036)	0.077
Q3	6.78–9.76	0.603 (0.420,0.865)	0.007	0.627 (0.414,0.952)	0.029	0.620 (0.407,0.942)	0.026
p for trend			0.006		0.026		0.024
**Log (VB12) (mcg)**							
Q1	−5.30–1.11	1		1		1	
Q2	1.11–2.42	0.897 (0.683,1.179)	0.43	0.815 (0.582,1.141)	0.228	0.819 (0.581,1.155)	0.248
Q3	2.42–11.00	0.586 (0.419,0.820)	0.002	0.633 (0.452,0.887)	0.009	0.635 (0.449,0.898)	0.011
p for trend			0.002		0.008		0.011
**Log (VC) (mg)**							
Q1	−2.30–3.55	1		1		1	
Q2	3.55–5.00	0.831 (0.624,1.108)	0.204	0.952 (0.682,1.329)	0.77	0.916 (0.653,1.285)	0.604
Q3	5.00–9.64	0.589 (0.422,0.823)	0.002	0.697 (0.471,1.033)	0.071	0.664 (0.450,0.980)	0.04
p for trend			0.002		0.076		0.042
**Log (VD) (mcg)**							
Q1	−3.00–0.77	1		1		1	
Q2	0.77–2.56	0.847 (0.649,1.105)	0.217	0.769 (0.555,1.064)	0.111	0.760 (0.545,1.059)	0.103
Q3	2.56–7.19	0.707 (0.505,0.990)	0.044	0.782 (0.544,1.124)	0.179	0.739 (0.517,1.059)	0.097
p for trend			0.041		0.161		0.087
**Log (VE) (mg)**							
Q1	−2.81–1.67	1		1		1	
Q2	1.67–2.42	0.749 (0.553,1.014)	0.061	0.839 (0.598,1.178)	0.305	0.854 (0.606,1.204)	0.359
Q3	2.42–5.04	0.472 (0.318,0.699)	< 0.001	0.506 (0.333,0.768)	0.002	0.519 (0.345,0.780)	0.002
p for trend			< 0.001		0.002		0.002
**Log (VK) (mcg)**							
Q1	−0.36–3.93	1		1		1	
Q2	3.93–4.99	0.750 (0.556,1.013)	0.06	0.700 (0.496,0.989)	0.043	0.703 (0.495,0.999)	0.049
Q3	4.99–10.72	0.486 (0.330,0.715)	< 0.001	0.560 (0.355,0.885)	0.014	0.564 (0.359,0.887)	0.014
p for trend			< 0.001		0.013		0.014

Data are presented as OR (95% CI) unless otherwise indicated. Model 1, without adjustment. Model 2, adjusted for gender, age, ethnicity, educational level, marriage, PIR and BMI. Model 3, adjusted for model 2 variables as well as the disease history (diabetes, hypertension, and malignancy), smoking status, alcohol drinking status, and physical activity.

**Figure 2:**
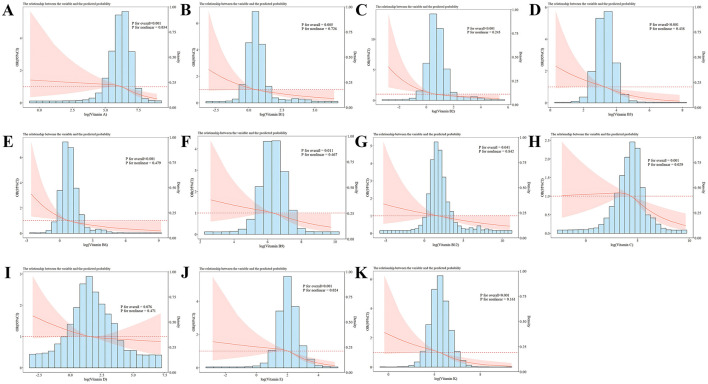
RCS analysis of the vitamin intake and sarcopenia risk with a 3-knot restricted cubic spline model based on Model 3. **(A–K)** Represented the association of vitamin A, vitamin B1, vitamin B2, vitamin B3, vitamin B6, vitamin B9, vitamin B12, vitamin C, vitamin D, vitamin E, vitamin K, and sarcopenia, respectively. Odds ratios were standardized to 1.00 for median vitamin intake. The red line represented the trend, and the red area was the 95% confidence interval. P for overall assessed the model's explanatory power, and P for non-linear signified the significance of the non-linear association.

The dose-response association of vitamin levels with the risk of sarcopenia in the study population was probed (Figure 2) in the RCS analysis. After multivariable adjustment, no notable relationship was found between VD levels and the incidence of sarcopenia (*P* for overall > 0.05), which is consistent with the results of logistic regression. Intake levels of vitamins A, C, and E exhibited a non-linear negative link to sarcopenia (*P* for overall < 0.001, P for non-linear < 0.05), while intake levels of vitamins B1, B2, B3, B6, B9, B12, and K showed a linear inverse relationship with sarcopenia (*P* for overall < 0.05, P for non-linear > 0.05).

### 3.3 Subgroup analysis and interaction

**Figure 3:**
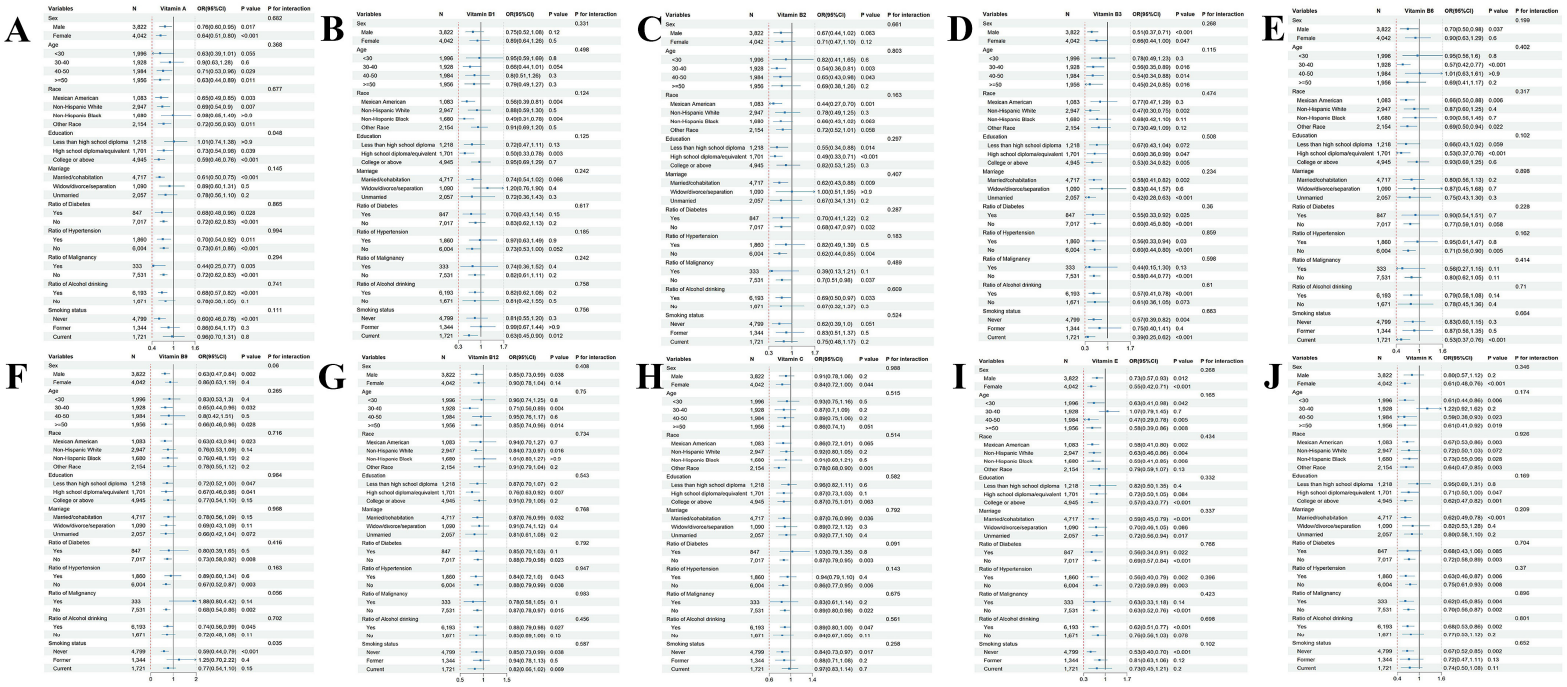
Subgroup analyses and interaction test of the association between vitamin intake and sarcopenia. **(A–J)** Represented the association of vitamin A, vitamin B1, vitamin B2, vitamin B3, vitamin B6, vitamin B9, vitamin B12, vitamin C, vitamin E, vitamin K, and sarcopenia, respectively.

Subgroup analysis was performed after adjustment for gender, age, ethnicity, education, marital status, PIR, BMI, medical history (hypertension, diabetes, and malignancy), smoking, drinking, as well as physical activity, for the evaluation of the link of vitamin intake to sarcopenia in different subgroups, and to explore potential interactions between vitamins and other variables (Figure 3). In the subgroup analysis by age, the intake of VA, VB3, VB9, VB12, VE, and VK in individuals aged 50 and older was negatively associated with the risk of sarcopenia (*P* < 0.05). In the subgroup analysis by gender, VA, VB3, VC, VE, and VK intake in females was negatively related to sarcopenia risk (*P* < 0.05), while in males, the intake of vitamins A, B3, B6, B9, B12, and E was negatively correlated with sarcopenia risk (*P* < 0.05). An interaction was observed between education and VA on the risk of sarcopenia (*P* for interaction = 0.048). In individuals with at least a high school education, a higher intake of VA was inversely correlated with the risk of sarcopenia (*P* < 0.05). In the diabetes subgroup analysis, higher intake of vitamins A, B3, and E was correlated with a significant sarcopenia risk reduction (*P* < 0.05). In the hypertension subgroup, increased intake of vitamins A, B3, B12, E, and K was notably related to a lower sarcopenia risk (*P* < 0.05). In the malignancy subgroup, VA and VK intake had a negative link to sarcopenia risk (*P* < 0.05). In the alcohol consumption subgroup, the relationship between higher vitamins A, B2, B3, B9, B12, C, E, and K intake and reduced sarcopenia risk was more pronounced (*P* < 0.05). For smokers, higher intake of vitamins A, B1, and B3 was notably linked to a lower risk of sarcopenia (*P* < 0.05).

### 3.4 Sensitivity analysis

A sensitivity analysis was carried out utilizing PSM, which led to the inclusion of 1,354 participants, with 677 in the sarcopenia group and 677 in the non-sarcopenia group. PSM adjusted for variables including gender, age, race, education, marital status, PIR, BMI, medical history, total caloric intake, smoking status, alcohol consumption, and physical activity, thereby minimizing the confounding differences between the sarcopenia and non-sarcopenia groups. Subsequently, three weighted logistic regression models were employed to investigate the potential associations between 11 vitamins and the risk of sarcopenia, as presented in Table 3. In Model 3, higher intakes of VA, VB2, VB6, and VC were significantly negatively associated with the risk of sarcopenia (OR < 1, *P* < 0.05). Specifically, in comparison to Q1, the Q3 levels of VA, VB2, VB6, and VC showed a negative correlation with the risk of sarcopenia [(VA, OR = 0.551, 95% CI: 0.368–0.824, *P* = 0.005), (VB2, OR = 0.612, 95% CI: 0.401–0.932, *P* = 0.023), (VB6, OR = 0.570, 95% CI: 0.369–0.880, *P* = 0.013), and (VC, OR = 0.639, 95% CI: 0.431–0.947, *P* = 0.027), respectively]. Other vitamins, including VB1, VB3, VB9, VB12, VD, VE, and VK, did not exhibit significant associations (*P* > 0.05).

**Table 3:** Multi-variate adjusted odds ratios (95% CIs) of risks of Sarcopenia in relation to different dietary vitamins levels among participants in NHANES 2011–2018 after propensity score matching (PSM).

**Variables**	**The range of dietary vitamin levels**	**Model 1**	***P*-value**	**Model 2**	***P*-value**	**Model 3**	***P*-value**
**Log (VA) (mcg)**							
Q1	−0.69–5.72	1		1		1	
Q2	5.72–6.64	0.575 (0.409,0.808)	0.002	0.558 (0.400,0.778)	< 0.001	0.521 (0.383,0.708)	< 0.001
Q3	6.64–8.90	0.691 (0.445,1.072)	0.097	0.658 (0.425,1.020)	0.061	0.551 (0.368,0.824)	0.005
p for trend			0.052		0.030		0.002
**Log (VB1) (mg)**							
Q1	−3.05–0.16	1		1		1	
Q2	0.16–0.90	1.288 (0.868,1.912)	0.204	1.260 (0.867,1.832)	0.220	1.212 (0.815,1.804)	0.333
Q3	0.90–6.46	0.963 (0.609,1.524)	0.871	0.964 (0.612,1.519)	0.873	0.846 (0.506,1.412)	0.513
p for trend			0.997		0.985		0.583
**Log (VB2) (mg)**							
Q1	−2.78–0.36	1		1		1	
Q2	0.36–1.12	0.824 (0.552,1.230)	0.338	0.797 (0.536,1.187)	0.258	0.725 (0.484,1.086)	0.116
Q3	1.12–5.36	0.752 (0.500,1.131)	0.168	0.708 (0.472,1.062)	0.093	0.612 (0.401,0.932)	0.023
p for trend			0.140		0.072		0.018
**Log (VB3) (mg)**							
Q1	0.32–2.96	1		1		1	
Q2	2.96–3.64	1.032 (0.680,1.568)	0.880	0.992 (0.651,1.513)	0.971	0.912 (0.586,1.418)	0.676
Q3	3.64–7.83	0.898 (0.586,1.377)	0.616	0.868 (0.565,1.334)	0.512	0.783 (0.481,1.275)	0.318
p for trend			0.651		0.523		0.316
**Log (VB6) (mg)**							
Q1	−2.59–0.39	1		1		1	
Q2	0.39–1.26	0.941 (0.627,1.411)	0.764	0.915 (0.598,1.401)	0.678	0.854 (0.543,1.344)	0.487
Q3	1.26–9.21	0.636 (0.428,0.946)	0.026	0.629 (0.412,0.959)	0.032	0.570 (0.369,0.880)	0.013
p for trend			0.033		0.036		0.013
**Log (VB9) (mcg)**							
Q1	2.60–5.89	1		1		1	
Q2	5.89–6.78	0.972 (0.615,1.539)	0.903	0.966 (0.618,1.508)	0.876	0.889 (0.570,1.387)	0.596
Q3	6.78–9.76	0.941 (0.597,1.485)	0.791	0.930 (0.594,1.456)	0.745	0.837 (0.529,1.324)	0.438
p for trend			0.792		0.742		0.428
**Log (VB12) (mcg)**							
Q1	−5.30–1.11	1		1		1	
Q2	1.11–2.42	0.871 (0.577,1.314)	0.504	0.853 (0.563,1.293)	0.447	0.793 (0.524,1.201)	0.266
Q3	2.42–11.00	0.719 (0.477,1.083)	0.112	0.729 (0.485,1.096)	0.126	0.676 (0.457,1.000)	0.050
p for trend			0.112		0.122		0.046
**Log (VC) (mg)**							
Q1	−2.30–3.55	1		1		1	
Q2	3.55–5.00	1.057 (0.723,1.545)	0.771	1.081 (0.731,1.600)	0.690	1.061 (0.713,1.578)	0.765
Q3	5.00–9.64	0.661 (0.450,0.970)	0.035	0.675 (0.457,0.997)	0.048	0.639 (0.431,0.948)	0.027
p for trend			0.044		0.059		0.034
**Log (VD) (mcg)**							
Q1	−3.00–0.77	1		1		1	
Q2	0.77–2.56	0.807 (0.511,1.274)	0.351	0.812 (0.519,1.268)	0.352	0.772 (0.493,1.208)	0.249
Q3	2.56–7.19	0.919 (0.567,1.490)	0.728	0.969 (0.614,1.529)	0.890	0.882 (0.565,1.376)	0.572
p for trend			0.681		0.806		0.498
**Log (VE) (mg)**							
Q1	−2.81–1.67	1		1		1	
Q2	1.67–2.42	1.347 (0.928,1.954)	0.115	1.326 (0.917,1.916)	0.130	1.218 (0.827,1.794)	0.309
Q3	2.42–5.04	0.926 (0.566,1.515)	0.755	0.888 (0.543,1.452)	0.630	0.778 (0.473,1.280)	0.315
p for trend			0.967		0.832		0.449
**Log (VK) (mcg)**							
Q1	−0.36–3.93	1		1		1	
Q2	3.93–4.99	0.901 (0.591,1.373)	0.621	0.887 (0.585,1.345)	0.566	0.841 (0.557,1.268)	0.399
Q3	4.99–10.72	0.791 (0.507,1.233)	0.295	0.783 (0.499,1.228)	0.280	0.733 (0.468,1.148)	0.170
p for trend			0.297		0.276		0.160

Data are presented as OR (95% CI) unless otherwise indicated. Model 1, without adjustment. Model 2, adjusted for gender, age, ethnicity, educational level, marriage, PIR and BMI. Model 3, adjusted for model 2 variables as well as the disease history (diabetes, hypertension, and malignancy), total caloric intake, smoking status, alcohol drinking status, and physical activity.

## 4 Discussion

In our study, the association between 11 vitamins and the incidence of sarcopenia was explored. Both non-linear and linear associations were identified between various vitamins and the occurrence of sarcopenia. Our findings suggest that higher intakes of VA(OR = 0.486), VB1 (OR = 0.582), VB2 (OR = 0.442), VB3 (OR = 0.569), VB6 (OR = 0.529), VB9 (OR = 0.620), VB12 (OR = 0.635), VC (OR = 0.664), VE (OR = 0.519), and VK (*OR* = 0.564) are notably related to a lowered incidence of sarcopenia. Specifically, VA, VC, and VE demonstrated a nonlinear negative association with sarcopenia, whereas vitamins B1, B2, B3, B6, B9, B12, and K exhibited a linear negative association. A notable association was not found between VD intake and the occurrence of sarcopenia. Our sensitivity analysis suggests that the results of negative correlations between VA, VB2, VB6, VC, and sarcopenia were more robust. This is the first large-scale study that comprehensively investigates the association of the intake of different vitamins with sarcopenia.

The association between VD and sarcopenia is a particularly intriguing topic. Several *in vitro* and animal studies have suggested that VD influences muscle cell proliferation, differentiation, and calcium homeostasis, playing a pivotal role in muscle physiology (23, 24). However, these findings are not always reflected in population-based studies. A Brazilian study involving 159 individuals aged 80 and above, which assessed VD intake through a 1-day dietary record (including supplements), showed no statistically significant association of VD intake with muscle mass, as measured by DXA (25), which aligns with our findings. Nevertheless, Swedish research of 719 individuals aged 70 found that VD intake, assessed via the diet history method, had a significantly positive association with the limb skeletal muscle mass index calculated via DXA (r = 0.219, *P* = 0.004), but no association was observed with walking speed or grip strength (26). A recent meta-analysis involving 15 studies and 2,866 participants aged 65 and above, examined the influence of VD on muscle strength and physical performance of the old in the community. The meta-analysis indicated that most studies reported no amelioration in muscle strength or physical performance after VD was supplemented either with or without calcium supplementation (27). The inconsistent conclusions across different population studies may arise from variations in study design, dosage, and baseline 25(OH)D levels in participants. Therefore, the relationship between VD and muscle health in the middle-aged and the old remains complex and is not fully defined, warranting further large-scale, rigorously designed studies.

As one person ages, the endogenous antioxidant defense system of the body is impaired and leads to an excessive accumulation of ROS that results in mitochondrial dysfunction and oxidative damage to muscle tissues. This process may directly or indirectly contribute to skeletal muscle atrophy (28, 29). Antioxidant vitamins, such as VC and VE, have been shown to prevent muscle protein oxidation and degradation, thereby helping to preserve muscle function and promote the antioxidant activity of glutathione (30, 31). Some observational studies indicate a strong association between VC intake and plasma VC levels with physical function and muscle mass in the old (32, 33), suggesting that adequate VC intake may help in the prevention of sarcopenia, a finding consistent with our results. Similarly, cross-sectional studies proved that higher VE intake is beneficial for muscle and skeletal health (30), with higher VE and fat intake linked to a lower incidence of sarcopenia (34). Further prospective research is necessary for examining the causal relationship between VC, VE, and the onset of sarcopenia. Few have delved into the connection of VA with sarcopenia. More research has focused on carotenoids, the main source of VA for the body, including α-carotene, β-carotene, γ-carotene, lycopene, lutein, zeaxanthin, and cryptoxanthin. Carotenoids, VC, and VE are collectively considered the most important antioxidants in the diet (32). A prospective study has shown that higher carotenoid intake in middle-aged and old individuals [age (61 ± 9 years)] is associated with increased grip strength and walking speed (35). In this study, VA was quantified in terms of retinol activity equivalents, providing a novel approach for future research. Reports on the function of VK in skeletal muscle are limited. To date, only a few studies demonstrated that VK helps to protect skeletal muscle. Research indicates the role of VK in modulating energy metabolism in skeletal muscle. VK-2 (MK4) has been shown to improve electron transfer in mitochondria and increase ATP production (36). It also helps maintain mitochondrial balance in skeletal muscle, a critical factor in preventing sarcopenia (37). Recently, a study based on NHANES explored the combined intake of vitamins A, C, E, and K as an antioxidant vitamin nutritional pattern and its effects on sarcopenia. The results demonstrated that the combined intake of these vitamins was significantly correlated with a reduced prevalence of sarcopenia, suggesting that this nutritional pattern could offer a new strategy for preventing muscle mass loss. Further prospective research is needed (13).

B vitamins, particularly VB1, VB3, VB6, VB9, and VB12, not only serve as cofactors in muscle synthesis but are also regarded as neurotrophic agents involved in bioenergetic and nutritional pathways. Current research predominantly focuses on the association between VB6, VB9, VB12, and sarcopenia. Elevated levels of homocysteine have been linked to a decline in muscle strength and gait speed, whereas vitamins B6, B9, and B12 have been shown to reduce serum homocysteine levels, thereby potentially offering benefits in sarcopenia prevention and control. A Dutch study evaluated the dietary habits and use of nutritional supplements among 227 old individuals (≥65 years) in the community via a food frequency questionnaire. The intake of VB6 and VB9 was markedly lower by 10%−18% in old individuals with sarcopenia compared with controls (*P* < 0.05) (20). Another case-control study included 66 old individuals with sarcopenia [mean age (71 ± 4 years)] and 66 age- and gender-matched controls and showed that the sarcopenia group had a 22% lower VB12 intake and 15% lower serum VB12 concentrations than the control cohort (*P* < 0.05 and *P* = 0.015) (11). Recently, one study based on the NHANES database investigated the link of VB1 and B2 intake to early-onset sarcopenia in American adults, revealing that higher intakes of VB1 and B2 may reduce the risk of developing early-onset sarcopenia (14), which aligns with our findings.

Our subgroup analysis also yielded some novel findings. The results of the age subgroup analysis indicate that, with increasing age, the number of vitamin types negatively correlated with sarcopenia increases. In the population under 30, only the intake of VE (*P* = 0.042) and VK (*P* = 0.006) showed a negative correlation with sarcopenia prevalence. However, in individuals aged 50 and above, the intake of VA (*P* = 0.011), VB3 (*P* = 0.016), VB9 (*P* = 0.028), VB12 (*P* = 0.014), VE (*P* = 0.008), and VK (*P* = 0.019) were all negatively correlated with sarcopenia. Age-related physiological changes, such as declines in digestive and absorption functions, increased risk of chronic diseases, and multiple vitamin deficiencies, may be significant contributing factors to this phenomenon. Future longitudinal studies are necessary to clarify the causal relationship between vitamin intake and sarcopenia and to optimize dietary recommendations for the old population. The diabetes subgroup analysis indicated that higher intakes of VA (*P* = 0.028), VB3 (*P* = 0.025), and VE (*P* = 0.022) notably reduced the risk of sarcopenia (*P* < 0.05). This finding differs from that of a previous prospective study and may be due to variations in the participant age and methods used to assess muscle mass. This study included 197 diabetic patients aged ≥65 years, with 93 individuals exhibiting a decline in muscle mass during an average 13.7-month follow-up. The intake of VB1 (*P* = 0.031), VB12 (*P* = 0.049), and VD (*P* = 0.004) was markedly lower than in those without muscle mass decline (38). In our baseline data, a statistically significant difference was not found in the proportion of men and women with sarcopenia, which contradicts previous studies (39, 40). This discrepancy may arise because prior studies included participants aged over 60 years, whereas our study included individuals aged under 60, a period during which estrogen continues to influence the metabolism and function of skeletal muscles in females (41). Notably, our gender-specific subgroup analysis revealed distinct protective effects: VB6, VB9, and VB12 exhibited stronger protective effects in men, while VC and VK had more pronounced protective effects in women. The specific mechanisms underlying these gender differences warrant further investigation, but these findings provide new references for developing gender-specific strategies for sarcopenia prevention. Sensitivity analysis demonstrates that the results showing an inverse correlation between the intake of vitamins VA, VB2, VB6, and VC and sarcopenia are more robust, likely due to propensity score matching, which better controlled for confounding bias between the two groups. Additionally, the inclusion of total caloric intake as a confounding factor further verified these findings. Future prospective studies with larger sample sizes are necessitated to elucidate the relationship between the intake of other vitamins and sarcopenia.

In our study, patients were at the age of 20–59, while sarcopenia is typically considered an age-related condition. Recent studies have revealed that some individuals begin to experience muscle wasting in midlife or even in their youth (42–44). This early-onset muscle loss is referred to as early-onset sarcopenia (45–47). Clinically, early-onset sarcopenia is associated with a poorer prognosis compared to typical sarcopenia (48). Patients with early-onset sarcopenia represent a unique high-risk group, and preventive and intervention strategies can effectively delay disease progression, thereby alleviating the burden on both patients and the healthcare system (48). In addition to nutritional interventions, exercise is a key measure in preventing sarcopenia. Resistance training, based on strength exercises, has been a proven safe and effective method. Individualized and periodic resistance training programs can enhance muscle strength, help maintain muscle mass, and counteract the progression of sarcopenia (49). The primary variables to consider when designing a resistance training program include training frequency, exercise selection, intensity, volume, and rest periods. It is recommended to perform training twice a week, combining upper and lower body exercises with a relatively high intensity for 1–3 sets of 6–12 repetitions per set (50).

Our study is the largest investigation to date into the relationship between the levels of 11 vitamins and the risk of sarcopenia, accounting for numerous potential confounding factors. Moreover, the majority of prior studies have focused on old populations, who are particularly vulnerable to malnutrition, oxidative stress, and declines in neurological integrity. In contrast, our study encompasses a nationally representative sample of Americans whose ages vary from 20 to 59, providing valuable insights for customizing vitamin intake based on life stages and conditions. However, our study does have certain limitations. First, it is cross-sectional and conducted at a single center, thus precluding the establishment of a causal relationship between vitamin levels and sarcopenia, so prospective cohort studies should be performed to confirm these results. Additionally, the sensitivity analysis shows that the results are not robust for all vitamins. Future studies with larger sample sizes or prospective designs are needed to further clarify this.

## 5 Conclusion

In conclusion, within a nationally representative group of American adults, the associations of the levels of 11 vitamins with the risk of sarcopenia were elucidated. In the future, researchers should elucidate whether interventions targeting the combination of vitamins, with or without other nutrients, can increase muscle mass in middle-aged and older populations, decrease the prevalence of sarcopenia, and ameliorate the life of sarcopenia sufferers.

## Data Availability

The original contributions presented in the study are included in the article/supplementary material, further inquiries can be directed to the corresponding author.
